# Oral Health of Parkinson's Disease Patients: A Case-Control Study

**DOI:** 10.1155/2018/9315285

**Published:** 2018-05-08

**Authors:** Marjolein A. E. van Stiphout, Johan Marinus, Jacobus J. van Hilten, Frank Lobbezoo, Cees de Baat

**Affiliations:** ^1^Foundation for Oral Health and Parkinson's Disease, P.O. Box 1155, 2340 BD Oegstgeest, Netherlands; ^2^Department of Neurology, Leiden University Medical Center, Albinusdreef 2, 2333 ZA Leiden, Netherlands; ^3^Department of Oral Kinesiology, Academic Centre for Dentistry Amsterdam (ACTA), University of Amsterdam and Vrije Universiteit Amsterdam, Gustav Mahlerlaan 3004, 1081 LA Amsterdam, Netherlands; ^4^Department of Dentistry, Radboud university medical center, P.O. Box 9101, 6500 HB Nijmegen, Netherlands

## Abstract

The aim of the study was to examine the oral health status of Parkinson's disease (PD) patients, to compare their oral health status to that of a control group, and to relate it to the duration and severity of PD. *Materials and Methods*. 74 PD patients and 74 controls were interviewed and orally examined. Among PD patients, the duration and the Hoehn and Yahr stage (HY) of the disease were registered. *Results*. More PD patients than controls reported oral hygiene care support as well as chewing/biting problems, taste disturbance, tooth mobility, and xerostomia, whereas dentate patients had more teeth with carious lesions, tooth root remnants, and biofilm. Both longer duration and higher HY were associated with more chewing problems and, in dentates, more teeth with restorations. In dentates, longer duration of the disease was associated with higher number of mobile teeth. Higher HY was associated with more oral hygiene care support as well as biting problems and, in dentates, more teeth with carious lesions and tooth root remnants. *Conclusions*. Comparatively, PD patients had weakened oral health status and reduced oral hygiene care. Both duration and severity of the disease were associated with more oral health and hygiene care problems.

## 1. Introduction

Parkinson's disease is a progressive degenerative neurological disorder, characterized by motor and nonmotor symptoms. The motor symptoms include akinesia, bradykinesia, rigidity, and tremor, which remain not restricted to the trunk and extremities, but may also occur in the orofacial system [1–3]. Motor impairments of the orofacial system include dysphagia, masticatory dysfunction, orofacial dyskinesia, and oromandibular dystonia [4–7]. In addition, related to oral health, the potentially impaired dexterity of arms and fingers may hamper the required daily oral hygiene care [8].

Advances in oral health care and treatment during the past few decades have resulted in a reduced number of edentulous individuals. The proportion of adults who retain their teeth until late in life has increased substantially [9]. Consequently, a still increasing number of dentate older people experience oral health problems, such as dental caries, periodontal disease, and substantial wear of hard tooth tissues (tooth wear). Furthermore, many older people have been treated with oral implants and/or sophisticated tooth- and/or implant-supported fixed and/or removable dental prostheses. Hence, these older people are in continuous need of both preventive and curative oral health care. The complexity of oral health status, the potential presence of systemic diseases, and the use of several medications make older people more vulnerable to oral problems when compared to younger age groups, particularly in those who are cognitively impaired [10, 11]. In addition, weakened oral health due to neglected oral hygiene care and reduced oral health care utilization has previously been found in older people [11–14].

Oral diseases, such as dental caries and periodontal disease, not only have oral effects, for example oral pain and oral functioning problems, but may also impact a number of systemic conditions. Emerging evidence suggests that poor oral health influences the initiation and/or progression of diseases, such as atherosclerosis, diabetes mellitus, Alzheimer's disease, and rheumatoid arthritis [15]. Aspiration of oropharyngeal bacteria may cause pneumonia [15–17]. Concerns were expressed about relationships between older people's poor oral health status and nutrition [18].

Study of the international literature revealed that, when compared to control subjects, Parkinson's disease patients generally had a lower number of teeth, more dental carious lesions, poorer periodontal health, higher objective periodontal treatment needs, more subjective chewing difficulties, more subjective swallowing difficulties, more subjective denture discomfort, more limited active mouth opening, and more negative impact of oral health on daily life (Table 1) [19–28]. However, each of the aforementioned studies investigated only few aspects of oral health; none investigated the whole picture of the oral health status. Furthermore, the relationships between aspects of oral health and the duration and severity of Parkinson's disease have not been addressed.

Therefore, the aim of the current study was to examine the most relevant aspects of the subjective and objective oral health status of Parkinson's disease patients, to compare their oral health status to that of an optimally gender-, age-, social background-, and lifestyle-matched control group, and to relate their oral health status to the duration and severity of Parkinson's disease.

## 2. Materials and Methods

### 2.1. Study Population

The current cross-sectional, case-control, optimally gender-, age-, social background-, and lifestyle-matched study was approved by the Medical Ethical Committee of Leiden University Medical Center, Leiden, the Netherlands, approval number P13.079. Assuming a power (1-*β*) of 0.80 and an *α* of 0.05 and an objective to detect a prevalence difference of 25% between groups across a range of different hypothetical prevalence rates, a sample size calculation indicated that 69 persons per group of Parkinson's disease patients and control subjects would be sufficient.

Patients with Parkinson's disease, without severe comorbidity according to classes III and IV of the Physical Status Classification System of the American Society of Anesthesiologists, were requested to participate when they visited the Department of Neurology of the Leiden University Medical Center, Leiden, the Netherlands, for a routine periodic consultation. The Parkinson's disease patients who agreed to participate, were subsequently requested to identify a control person, for instance a family member or other close relative, who had no Parkinson's disease or other severe systemic diseases according to classes III and IV of the Physical Status Classification System of the American Society of Anesthesiologists, who had approximately the same age (±5 years) as well as a similar social background and lifestyle, and who would likely be prepared to participate. The group of control subjects was also optimally gender matched, meaning that men with Parkinson's disease preferably indicated men and women with Parkinson's disease preferably indicated women. Assuming that not every person proposed by a Parkinson's disease patient as control subject would agree to participate, initially 74 Parkinson's disease patients were included. All Parkinson's disease patients and indicated control subjects were visited at their homes to inform them about the research project. Luckily, all of them provided informed consent and were subsequently interviewed and examined.

After the interview and the examination, every participant received information on his/her actual oral health condition and was recommended consultation with a dentist in case the actual oral health condition required attention and/or treatment.

### 2.2. Assessments

Using a common history form, data were gathered about educational level (primary, secondary, and tertiary), smoking habits, length of time since the last oral health consultation, number of oral health consultations during the previous five years, daily oral hygiene care (whether or not supported by a professional or voluntary care provider), type of toothbrush used, chewing problems, biting problems, taste disturbance, burning mouth, xerostomia, halitosis, remaining food particles, tooth mobility, toothache, tooth sensitivity, painful gums, and bleeding gums. Persons with an edentulous maxilla/mandible were requested to indicate the duration since the last teeth in the maxilla/mandible had been removed, the number of years during which a current complete maxillary/mandibular removable dental prosthesis was functioning, and their potential experience with a loose coming complete maxillary/mandibular removable dental prosthesis during oral movements.

An experienced dentist performed an oral health examination in all participants, using a common oral screening form. Variables included were edentulousness, soft tissue lesions, complete or partial maxillary/mandibular removable dental prostheses, number of teeth, number of teeth with carious lesions, number of teeth with restorations, number of tooth root remnants, amount of biofilm and food, periodontal health, and number of posterior functional tooth units, including (implant-supported) single- and multiunit fixed dental prostheses.

The amount of biofilm and food on teeth and soft tissues was assessed by a simple 3-points scale: 1 = hardly any biofilm and food; 2 = thin layer of biofilm and food; 3 = thick layer of biofilm and food.

Periodontal health was assessed using the tooth mobility scoring system. This clinically easy-to-determine system differentiates three grades: grade I: mobility in a horizontal direction more than 0.2 mm and less than 1 mm; grade II: mobility in a horizontal direction of 1 mm or more; and grade III: mobility in vertical direction [29].

The number of posterior functional tooth units is an important proxy for masticatory efficiency. One maxillary and one mandibular premolar in occluding contact constitute one posterior functional tooth unit. One occluding maxillary and mandibular molar are equivalent to two posterior functional tooth units [30].

In persons with an edentulous maxilla/mandible, the reduction of the edentulous residual alveolar ridge was clinically classified as moderate reduction, high degree of reduction, or extensive reduction, using a standard set of edentulous alveolar ridge models [31].

For Parkinson's disease patients, the duration of the disease (since the onset of motor symptoms) and the severity of the disease expressed by the Hoehn and Yahr stage were registered from the patients' medical records [32]. The duration of the disease was categorized as less than 5 years, between 5 and 9 years, and 10 years or longer.

### 2.3. Statistical Analysis

Data were analyzed using SPSS version 22.0 (SPSS, Inc., Chicago, IL). Numbers and percentages were compared between groups using a Chi-square test (*χ*^2^). An independent-samples Student's *t*-test was only used to compare the age of Parkinson's disease patients and control subjects. Mann–Whitney *U* test was used to compare ordinal or nonnormally distributed continuous variables between groups. Kruskal–Wallis test was used to examine group differences of nonnormally distributed continuous variables with three or more categories. Statistical significance was accepted at *P* < 0.05. Given the exploratory character of the study, no attempt was made to control for multiple comparisons.

## 3. Results

### 3.1. Participants

Interviews and oral health examinations were performed in 26 women and 48 men with Parkinson's disease and in 35 female and 39 male control subjects (*χ*_(1)_^2^=2.259, *P*=0.133). Mean age ± standard deviation was 70.2 ± 8.8 years in the Parkinson's disease patients and 67.9 ± 10.1 years in the control subjects (Student's *t*-test; *P*=0.641).

### 3.2. Subjective Variables

Table 2 presents frequencies and percentages of the subjective variables of the Parkinson's disease patients and the control subjects. When compared to the control subjects, statistically significantly more Parkinson's disease patients reported daily oral hygiene care support by a professional or voluntary care provider, chewing problems, biting problems, taste disturbance, and xerostomia. When compared to the dentate control subjects, statistically significantly more dentate Parkinson's disease patients reported tooth mobility.

The Parkinson's disease patients and control subjects with an edentulous maxilla (and mandible) showed no statistically significant group differences with regard to length of time since the last teeth had been removed, number of years during which a current complete maxillary/mandibular removable dental prosthesis was functioning, and persons' experiences with a loose coming complete maxillary/mandibular removable dental prosthesis during oral movements.

### 3.3. Objective Variables

Table 3 presents frequencies and percentages of the objective variables of the Parkinson's disease patients and the control subjects. Statistical analysis of the data of dentate persons did point out that the Parkinson's disease patients had statistically significantly more teeth with carious lesions, a greater number of tooth root remnants, and a greater amount of biofilm and food when compared to the control subjects.

Only few Parkinson's disease patients and control subjects had teeth with grades II and III of tooth mobility, 11 and 6 persons, respectively. Therefore, comparisons of periodontal health between Parkinson's disease patients and control subjects were not performed.

The persons who had an edentulous maxilla/mandible, showed no statistically significant differences between Parkinson's disease patients and control subjects with regard to grades of reduction of the edentulous residual alveolar ridges.

### 3.4. Parkinson's Disease Patients

The distribution of the Parkinson's disease patients across duration and Hoehn and Yahr stage of the disease is presented in Table 4.

The mean duration of the disease was 9.1 ± 6.4 years. Reported chewing problems were statistically significantly positively related to the duration of the disease (*χ*_(8)_^2^=17.690, *P*=0.024). In dentate patients, the number of teeth with restorations and the number of teeth with mobility grade II or III were statistically significantly related to the duration of the disease (Kruskal–Wallis test; resp. *H*_(2)_=6.398, *P*=0.041 and *H*_(2)_=8.058, *P*=0.018).

For subsequent statistical analysis, the Hoehn and Yahr stages were dichotimized, resulting in a group of 47 patients with the mild stages 1 and 2 and a group of 27 patients with the moderate/severe stages 3, 4, and 5. The reported chewing and biting problems as well as the reported daily support for oral hygiene care by a professional or voluntary care provider were statistically significantly positively related to the Hoehn and Yahr stage of the disease (resp. *χ*_(4)_^2^=14.045, *P*=0.007; *χ*_(4)_^2^=10.939, *P*=0.027; *χ*_(1)_^2^=11.457, *P*=0.001). Furthermore, the number of teeth with carious lesions, the number of teeth with restorations, and the number of tooth root remnants appeared statistically significantly higher in dentate patients with the moderate/severe Hoehn and Yahr stages 3–5, when compared to dentate patients with the mild Hoehn and Yahr stages 1-2 (Mann–Whitney *U* test; resp., *U*=246.500, *P*=0.001; *U*=252.500, *P*=0.004; *U*=311.000, *P*=0.002).

## 4. Discussion

This is the first study which examined the most relevant aspects of the subjective as well as the objective oral health status of a large group of Parkinson's disease patients, which compared these findings with the same data of an optimally gender-, age-, social background-, and lifestyle-matched control group and which related the oral health status of the Parkinson's disease patients to the duration and severity of the disease. The findings demonstrate that more Parkinson's disease patients than control subjects reported daily oral hygiene care support by a professional or voluntary care provider, as well as chewing problems, biting problems, taste disturbance, tooth mobility, and xerostomia. Objectively, the dentate Parkinson's disease patients had a greater number of teeth with carious lesions, a greater number of tooth root remnants, and a greater amount of biofilm and food, when compared to the dentate control subjects. These findings represent symptoms of weakened oral health and reduced oral hygiene care, probably due to Parkinson's disease impairments. Within the group of Parkinson's disease patients, both longer duration and higher Hoehn and Yahr stage of the disease were associated with more chewing problems and, in dentate persons, with more teeth with restorations. Additionally, in dentate persons, longer duration of the disease was associated with a higher number of teeth with mobility grade II or III, whereas a higher Hoehn and Yahr stage of the disease was associated with more daily oral hygiene care support by a care provider as well as biting problems and, in dentate persons, with more teeth with carious lesions and more tooth root remnants. These findings reflect symptoms of weakening oral health, probably due to the reducing ability to manage oral hygiene care as the disease advances.

Existing data on the oral health of Parkinson's disease patients, as presented in Table 1, are extended by the results of the current study. Novel identified oral health problems include taste disturbance and more oral health problems in advanced stages of the disease. Together, these data indicate that weakening oral health and its potential negative impact on several systemic conditions are serious problems in Parkinson's disease patients, which demand more attention worldwide by the multidisciplinary Parkinson's disease medical management teams as well as standard referrals to oral health-care providers.

Chewing and biting problems, more reported by Parkinson's disease patients than control subjects, predominantly in advanced stages of the disease, may reflect (increasing) motor impairments of the orofacial system. Consequently, it is recommended to consider research of chewing and biting problems in Parkinson's disease patients with the objective to manage or reduce these problems. Other impairments of the orofacial system of Parkinson's disease patients may present as temporomandibular dysfunction. A recent study among a group of Parkinson's disease patients found temporomandibular dysfunction in about one-fifth of the patients [33]. Nevertheless, since diagnosing and classifying temporomandibular dysfunction is a rather complicated and time-consuming activity [34], we decided consciously not to include temporomandibular dysfunction as a research variable in our study. A separate and specific study on this topic is in preparation by the research groups involved in the current study.

When considered in relation to oral health, taste disturbance is certainly a novel finding in Parkinson's disease patients since none of the studies mentioned in Table 1 reported this problem. However, olfactory loss as well as smell and taste loss are well-known neurological problems in Parkinson's disease. Results of a recent (neurological) study suggest that the problems are caused by a decline of central brain networks rather than a damage of the peripheral olfactory system [35]. Previously, the olfactory deficit was demonstrated to be independent of Parkinson's disease severity and duration and preceding clinical motor symptoms by years. For this reason, taste disturbance was even suggested to be used for assessing the risk of Parkinson's disease in otherwise asymptomatic individuals [36]. From an oral health perspective, taste ability may change due to deterioration of oral health status, deficient oral hygiene, and impaired masticatory ability [37]. Additionally, saliva is of great importance since it acts as a solvent of taste substances, affects taste sensitivity, and maintains the health and function of the taste receptors. Consequently, hyposalivation results, among others, in significant altered taste sensation or taste disturbance [38]. Hyposalivation may induce oral health problems, such as tooth wear, oral soft tissue lesions, dental caries, candidiasis, and periodontal disease [39]. Nearly 65% of the Parkinson's disease patients in our study reported xerostomia (Table 2), confirming previous results demonstrating or suggesting that xerostomia and the commonly underlying hyposalivation are prevalent complications of Parkinson's disease [28, 40]. Another saliva complication of Parkinson's disease patients is drooling. Most likely, impaired intraoral saliva clearance is the basis of its pathophysiology. However, research to explore the exact pathophysiology and to develop standard diagnostic criteria and assessment tools are needed [41]. Therefore, taste disturbance, xerostomia, hyposalivation, and drooling are topics challenging collaboration between movement disorders specialists and dentists.

Several results of the current study suggest a reduced ability to manage oral hygiene care due to Parkinson's disease impairments, which increases as the disease advances. This assumption concurs with the finding of impaired dexterity in Parkinson's disease, predominantly in advanced stages of the disease [8]. Furthermore, a recent study proved that fine motor skills in Parkinson's disease patients are impaired, predominantly in patients with mild cognitive impairment [42]. Probably, at a certain, difficult to predict stage of Parkinson's disease, patients become dependent on professional or voluntary care providers for proper daily oral hygiene care. In the current study, 15% of the Parkinson's disease patients reported as such. Unfortunately, oral hygiene care is generally not prioritized, either by the professional care providers, or by the patients themselves. Even providing a guideline to nursing home care providers and supervised implementation of this guideline did not result in a general improvement of oral hygiene of nursing home residents [43]. Subsequently, it was recommended to better integrate professional oral hygiene care into professional general health care (also in Parkinson's disease patients) in order to prevent poor oral health to become a new geriatric syndrome [44].

A retrospectively ascertained weakness of this study is the lack of data on social background and lifestyle of both the Parkinson's disease patients and the control subjects. Although the patients were requested to identify a family member or other close relative who had a similar social background and lifestyle as a control person, these variables were not actually assessed. Therefore, some selection bias cannot be ruled out.

## 5. Conclusions

The results of the current study reveal that the Parkinson's disease patients had a weakened oral health status and reduced oral hygiene care, when compared to an optimally gender-, age-, social background-, and lifestyle-matched control group. Additionally, both longer duration of the disease and more severe disease were associated with more oral health and oral hygiene care problems, altogether suggesting that their weakened oral health and reduced oral hygiene care are due to Parkinson's disease impairments. The authors recommend worldwide multidisciplinary Parkinson's disease medical management teams to pay more attention to their patients' oral health including standard referrals to oral health-care providers, to establish research of chewing and biting problems, taste disturbance, xerostomia, hyposalivation, and drooling in Parkinson's disease patients through collaboration of movement disorders specialists and dentists, and to integrate professional oral hygiene care into professional general health care for Parkinson's disease patients.

## Figures and Tables

**Table 1:** Studies on Parkinson's disease and oral health, available in the international literature.

Publication	Country	Research design	Population	Results of PD patients when compared to controls	OR	95% CI	*P*
Nakayama et al., 2004 [19]	Japan	Questionnaire survey by mail	104 with PD 191 controls	Gender- and age-adjusted:			
				More chewing difficulties	6.0	2.8–12.8	
				More denture discomfort	3.9	1.9–8.0	
				More edentulousness	3.5	1.8–6.8	
				Less daily denture care	10.5	2.9–37.3	
				50% swallowing problems			

Schwarz et al., 2006 [20]	Germany	Case-control, age-matched	70 with PD 85 controls	Higher scores on indices of the Community Periodontal Index for Treatment Needs (CPITN)			<0.05

Einarsdóttir et al., 2009 [21]	Iceland	Case-control	67 with PD 55 controls	Lower number of teeth			<0.036
				More dental carious lesions			<0.007
				More biofilm	3.13	1.4–6.9	<0.004
				Poorer periodontal health	2.28	1.0–4.9	0.035
				Greater number of cariogenic bacteria in saliva			<0.05

Hanaoka and Kashihara, 2009 [22]	Japan	Case-control, age-matched	89 with PD 68 mild cognitively impaired 60 with ischemic stroke	Lower number of teeth			<0.05
				More dental carious lesions			<0.001
				More deep periodontal			<0.001
				pockets			

Bakke et al., 2011 [23]	Denmark	Case-control, age-matched, gender-matched	15 with moderate to advanced PD 15 controls	Overall objective orofacial function			<0.001
				Poorer subjective masticatory ability			<0.001
				Poorer active mouth opening			<0.001
				More negative impact of oral health on daily life			<0.001

Müller et al., 2011 [24]	Germany	Case-control	101 with PD 75 controls	Lower gingival index			<0.001
				Lower frequency of daily tooth brushing			<0.01
				More dental carious lesions			<0.01
				Longer time since last dental visit			<0.001
				Lower salivary flow rate			<0.001
				More gingival recession			<0.001
				More tooth mobility			<0.001

Cicciù et al., 2012 [25]	Italy	Case-control, age-matched	45 with mild to moderate PD 45 controls	More dental carious lesions			not reported
				Higher gingival index			not reported
				Higher sulcus bleeding index			not reported
				Higher biofilm index			not reported

Pradeep et al., 2015 [26]	India	Case-control, age-matched	45 with PD 46 controls	More periodontal pockets			<0.001
				More periodontal attachment loss			<0.001
				Lower gingival index			<0.001
				Lower biofilm index			<0.001

Ribeiro et al., 2016 [27]	Brasil	Case-control	Wearers of complete removable dental prostheses 17 with PD 20 controls	Poorer self-perception of oral health			<0.04

Barbe et al., 2017 [28]	Germany	Questionnaire survey	100 with PD Frequencies compared with results of other studies	Poorer oral health impact profile, among others due to complaints of xerostomia, drooling and dysphagia			

**Table 2:** Frequencies, including percentages, of the general subjective aspects of oral health and the often/occasional oral health complaints of the (dentate) Parkinson's disease patients (PD) and the (dentate) control subjects (control) and the results of the Chi-square test carried out to assess statistically significant differences (∗) between PD and control.

Variables	PD	Control	Chi-square test
*All persons: general subjective variables*	*n*=74	*n*=74	
Educational level			
(i) primary	18 (24%)	12 (16%)	
(ii) secondary	21 (29%)	35 (47%)	
(iii) tertiary	34 (46%)	27 (37%)	
(iv) missing value	1 (1%)	—	*χ*_(7)_^2^=11.947; *P*=0.102
Smoking status	6 (8.1%)	6 (8.1%)	—
Length of time since the last oral health consultation			
(i) less than half a year	52 (70.3%)	49 (66.2%)	
(ii) between a half and two years	15 (20.3%)	22 (29.8%)	*χ*_(5)_^2^=5.704; *P*=0.336
Number of oral health consultations during the previous five years			
(i) 0	4 (5.4%)	2 (2.7%)	
(ii) 1–5	13 (17.6%)	17 (23.0%)	
(iii) 6–10	30 (40.5%)	36 (48.6%)	
(iv) 11 or more	27 (36.5%)	19 (25.7%)	*χ*_(6)_^2^=6.607; *P*=0.359
Daily oral hygiene care supported by a professional or voluntary care provider	11 (14.9%)	1 (1.4%)	*χ*_(1)_^2^=9.069; *P*=0.003^∗^
Electric toothbrush used	36 (48.6%)	30 (40.5%)	*χ*_(3)_^2^=3.091; *P*=0.378

*All persons: oral health complaints*	*n*=74	*n*=74	
Chewing problems	22 (29.7%)	3 (4.1%)	*χ*_(4)_^2^=18.973; *P*=0.001^∗^
Biting problems	26 (35.1%)	7 (9.5%)	*χ*_(4)_^2^=15.047; *P*=0.005^∗^
Taste disturbance	17 (23.0%)	1 (1.4%)	*χ*_(4)_^2^=19.523; *P*=0.001^∗^
Burning mouth	3 (4.1%)	0	*χ*_(4)_^2^=8.0290; *P*=0.091
Xerostomia	48 (64.9%)	24 (32.4%)	*χ*_(4)_^2^=19.510; *P*=0.001^∗^
Halitosis	14 (18.9%)	9 (12.2%)	*χ*_(4)_^2^=7.037; *P*=0.134
Remaining food particles	52 (70.3%)	51 (68.9%)	*χ*_(4)_^2^=2.877; *P*=0.579

*Dentate persons: oral health complaints*	*n*=65	*n*=65	
Tooth mobility	12 (18.5%)	2 (3.1%)	*χ*_(3)_^2^=11.215; *P*=0.011^∗^
Toothache	10 (15.4%)	6 (9.2%)	*χ*_(3)_^2^=2.000; *P*=0.572
Tooth sensitivity	17 (26.2%)	11 (16.9%)	*χ*_(4)_^2^=4.500; *P*=0.343
Painful gums	12 (18.5%)	7 (10.8%)	*χ*_(4)_^2^=2.810; *P*=0.590
Bleeding gums	13 (20.0%)	12 (18.5%)	*χ*_(4)_^2^=5.826; *P*=0.213

**Table 3:** Frequencies, including percentages, of the objective oral health variables of the Parkinson's disease patients (PD) and the control subjects (control) and statistically significant group differences.

Variables	PD	Control	Statistical test
*All persons*	*n*=74	*n*=74	
Number of persons with an edentulous maxilla	14 (18.9%)	14 (18.9%)	
Number of persons with an edentulous maxilla and mandible	9 (12.2%)	9 (12.2%)	
Number of persons with a soft tissue lesion	20 (27.0%)	18 (24.3%)	
Number of complete maxillary removable dental prostheses	14	15	
Number of complete mandibular removable dental prostheses	9	9	
Number of partial maxillary removable dental prostheses	8	7	
Number of partial mandibular removable dental prostheses	10	9	

*Dentate persons*	*n*=65	*n*=65	
Mean number of teeth	21.2	22.5	
Number of teeth with carious lesions	74	12	Mann–Whitney *U* test; *U*=1526.500, *P* ≤ 0.001
Number of teeth with restorations	466	518	
Number of tooth root remnants	24	5	Mann–Whitney *U* test; *U*=1818.000, *P* < 0.022
Amount of biofilm and food (scores 2 and 3)	39 (60%)	20 (31%)	*χ*_(2)_^2^=18.127; *P* < 0.001
Mean number of posterior functional tooth units, including (implant-supported) single- and multiunit fixed dental prostheses	3.2	2.8	

**Table 4:** Distribution, including percentages, of the Parkinson's disease patients by duration (D) and Hoehn & Yahr stage (HY) of the disease.

D/HY	Number of patients	Percentage
D less than 5 years	20	27
D between 5 and 9 years	19	26
D 10 years or more	35	47
HY1	16	22
HY2	31	42
HY3	11	14
HY4	12	16
HY5	4	6

## Data Availability

The data used to support the findings of this study are available from the corresponding author upon request.
